# A Five-Year Single-Center Retrospective Study of Neoadjuvant Therapy Response and Survival in Romanian Women with Breast Cancer

**DOI:** 10.3390/life16040613

**Published:** 2026-04-07

**Authors:** Adeline-Roxana Bucur, Alexandru Cristian Cindrea, Antonia Armega-Anghelescu, Alin Marian Vasile, Octavian Constantin Neagoe, Paul Szeica, Ovidiu Alexandru Mederle, Flavia Zara

**Affiliations:** 1 Doctoral School, “Victor Babes” University of Medicine and Pharmacy, E. Murgu Square, No. 2, 300041 Timisoara, Romania; adeline.bucur@umft.ro (A.-R.B.); antonia.armega@umft.ro (A.A.-A.); alin.vasile@umft.ro (A.M.V.); paul.szeica@umft.ro (P.S.); 2 Department of Pathology, Emergency City Hospital, 300254 Timisoara, Romania; flavia.zara@umft.ro; 3 Emergency Department, Emergency Clinical Municipal Hospital, 300254 Timisoara, Romania; mederle.ovidiu@umft.ro; 4 Department of Microscopic Morphology, “Victor Babes” University of Medicine and Pharmacy, 300041 Timisoara, Romania; 5 Second Clinic of General Surgery and Surgical Oncology, Emergency Clinical Municipal Hospital, 300041 Timisoara, Romania; dr.octavian.neagoe@gmail.com; 6 Second Discipline of Surgical Semiology, First Department of Surgery, “Victor Babes” University of Medicine and Pharmacy, 300041 Timisoara, Romania; 7 Breast Surgery Research Center, “Victor Babes” University of Medicine and Pharmacy, 300079 Timisoara, Romania; 8 Department of Surgery, “Victor Babes” University of Medicine and Pharmacy, 300041 Timisoara, Romania; 9 Angiogenesis Research Center, “Victor Babes” University of Medicine and Pharmacy, E. Murgu Square, No. 2, 300041 Timisoara, Romania

**Keywords:** mammary gland, tumor morphology, therapy response, chemotherapy

## Abstract

**Background**: Breast cancer remains a major public health problem, with increasing incidence and persistent survival disparities. In Romania, barriers to early diagnosis and access to multidisciplinary treatment may contribute to poorer outcomes. **Methods**: We conducted a retrospective single-center cohort study including 118 women diagnosed with and/or treated for breast cancer in our institution between 1 January and 31 December 2020. Patients were followed for 5 years. The primary outcome was overall survival (OS). Clinicopathological characteristics, treatment exposure, pathological response after neoadjuvant therapy, and factors associated with OS were analyzed. **Results**: The median age at diagnosis was 62 years. Most tumors were located in the upper quadrants, and the most frequent subtype was hormone receptor-positive/HER2-negative breast carcinoma. During follow-up, 26.27% of patients died from disease progression or associated complications. Estimated OS was 88.7% at 1 year and 72.8% at 5 years. Older age at diagnosis and treatment exposure patterns, including the absence of neoadjuvant therapy, were associated with OS. **Conclusions**: In this single-center retrospective cohort, overall survival was associated with age at diagnosis, tumor characteristics, and treatment patterns. The high proportion of early deaths and the frequent absence of documented surgical treatment in patients who died suggest important challenges related to late presentation, continuity of care, and access to guideline-concordant multidisciplinary treatment in the Romanian setting.

## 1. Introduction

Breast cancer is recognized worldwide as the leading cause of death by cancer in women [1], with its incidence increasing at various rates across the globe [2]. In Romania, breast cancer accounts for 26.9% of all female cancer diagnoses, with an increasing incidence [3], and a mortality rate of 31 deaths per 100,000 population, 2% higher than the EU average [4]. Several socioeconomic factors may contribute to this increased mortality, particularly the absence of standardized screening programs and late presentation to medical care [5].

Although it affects primarily women aged over 50 years old, the number of cases of early-onset neoplasia is rising [6]. Nevertheless, women aged 65 years and older had a mortality rate 10 times higher than that of younger women, whereas for all cancers combined, the difference was 14-fold [7]. Breast cancer represents a heterogeneous group of histological entities, with invasive ductal carcinoma being the most frequent type [8].

Though the most common malignant breast tumors are carcinomas, other entities like melanoma, lymphoma, sarcoma, or metastases can develop. The spectrum of carcinomas varies from invasive ductal carcinoma no special type (NST) to the rare special types like salivary gland-like carcinomas, which are characterized by genetic alterations with therapeutic implications [9]. The further subclassification of carcinomas into molecular subgroups based on the hormone receptors, human epidermal growth factor receptor2 (HER-2) protein, and Ki67 immunophenotype is indispensable for the treatment and prognosis [10].

The four molecular categories are luminal A, defined as HER2-negative tumors expressing hormone receptors (HR) with a low proliferation index; luminal B, which includes either HR-positive/HER2-positive tumors or HR-positive/HER2-negative tumors with a high proliferation index; HER2-positive tumors, which are HR-negative; and triple-negative breast cancers (TNBC), which express neither hormone receptors nor HER2 [11]. The latest advanced research recognizes two other HER2 categories with clinical and therapeutic relevance: HER2-low breast cancer with low HER2 expression and ultra-low HER2 cancers, characterized by minimal expression of the protein [12].

The initial investigation of choice for the preoperative diagnosis of breast lesions is core needle (tru-cut) biopsy, as it provides a sufficient amount of tissue for histopathological diagnosis [13], followed by immunohistochemical assessment of estrogen receptor (ER), progesterone receptor (PR), HER2, and Ki-67 status [14], with additional HER2 genetic testing when required.

Tumor size, lymph node status, histological type, histological grade, and molecular subtype are well-known prognostic and predictive factors for breast cancers [15]. Current oncological management according to the National Comprehensive Cancer Network (NCCN) guidelines clearly defines candidates for preoperative (neoadjuvant) systemic therapy and adjuvant systemic therapy [16], with neoadjuvant systemic therapy being an important part of the individualized breast cancer treatment [17].

Surgical management of non-metastatic invasive breast cancer includes complete surgical excision of the primary tumor and locoregional staging of axillary lymph nodes. Axillary staging can be clinical, radiological, and surgical, by axillary lymph node dissection (ALND) and sentinel lymph node biopsy (SLNB) [18].

The evaluation of the tumor response to neoadjuvant chemotherapy in the surgical specimen is done using different systems, the ones recommended by the College of American Pathologists (CAP) being the American Joint Committee on Cancer (AJCC) and Residual Cancer Burden (RCB) systems [19]. The RCB method quantifies the extent of residual disease [20]. The luminal subtype of breast cancer shows a better long-term prognosis than that of HER2-enriched or triple-negative breast cancer [21].

Several clinical and pathological parameters have been used to determine breast cancer prognosis, including stage at diagnosis, histological grades, intrinsic subtypes, and metabolic comorbidities [22].

The primary aim of this study was to evaluate 5-year overall survival in a retrospective cohort of patients with breast cancer treated in a single tertiary center. Secondary aims were to examine the association of overall survival with age at diagnosis, tumor stage, histological grade, molecular subtype, surgical treatment, and the administration of neoadjuvant therapy, and to explore predictors of treatment response in patients who received neoadjuvant treatment.

## 2. Materials and Methods

### 2.1. Study Design

This retrospective single-center study included 118 women with breast cancer who were evaluated or admitted to the Surgical Oncology and General Surgery Departments of the Timișoara Municipal Hospital between 1 January 2020 and 31 December 2020 and were followed for 5 years. Patient data were obtained from the hospital computer database, and for the five-year survival, a report from the Population Register in Timisoara was requested.

All types of cancers were included in the study. Age at diagnosis, tumor location, types of interventions, neoadjuvant chemotherapy, tumor staging, histological type, grading, and molecular subtype were described.

Associations between variables were analyzed. The staging of the tumors was done according to the latest AJCC using the TNM (Tumor, Node, Metastasis) system, with further subdivision into pathological stages.

The carcinomas were divided in the following pathological stages: stage 0 (DCIS or ypT0 tumors), stage IA (pT1N0), stage IB (pT0-1N1mi), stage IIA (pT0-1N1, pT2N0), stage IIB (pT2N1, pT3N0), stage IIIA (pT0-2N2, pT3N1-2), stage IIIB (pT4N0-2), stage IIIC (anyTN3), stage IV (any T, any N, M1) [20].

The differentiation was assessed based on the Nottingham score, widely used in pathology reports as a standard of tumor grading. It’s a 3-tier system taking into consideration gland formation, nuclear pleomorphism, and mitotic activity [21].

The ER and PR are traditional prognostic and predictive factors in breast cancer, and both are the mainstays of gene expression profiles to determine intrinsic breast cancer subtypes. ER- or PR-positive breast cancers are classified as the luminal subtype, which have a more favorable prognosis and are more responsive to anti-estrogen therapy than that of ER- and PR-negative breast cancer [22].

In evaluating the response to therapy, the AJCC/TNM system was adopted, taking into consideration the largest contiguous focus of the invasive cancer in determining ypT stage in ypTNM system [23]. Neoadjuvant treatment in our institution followed the NCCN guideline recommendations in use at the time of diagnosis.

### 2.2. Data Collection

Patient data were collected from the hospital database, and five-year survival status was obtained from a report requested from the Population Register in Timișoara.

Data regarding patients’ demographics (sex, age, date of admission, and date of death), surgical procedures, tumor staging, histological grading, and molecular subtype were collected using Microsoft Excel (Microsoft Office 16, Albuquerque, NM, USA). Immunohistochemistry (IHC) results used to determine molecular subtype were available for 74 of the 117 carcinoma cases. For the remaining cases, molecular subtypes were recorded as unknown due to unavailable IHC data.

### 2.3. Inclusion and Exclusion Criteria

Inclusion criteria were histologically confirmed breast cancer and availability of sufficient clinicopathological information for analysis; for resection specimens, only cases with pathological staging were included. Exclusion criteria were frozen-section-only specimens and incomplete records. Two patients presented with metastatic disease at diagnosis. Follow-up was performed retrospectively for 5 years using the available hospital documentation and outcome records, and overall survival was evaluated during this interval.

**Figure 1:**
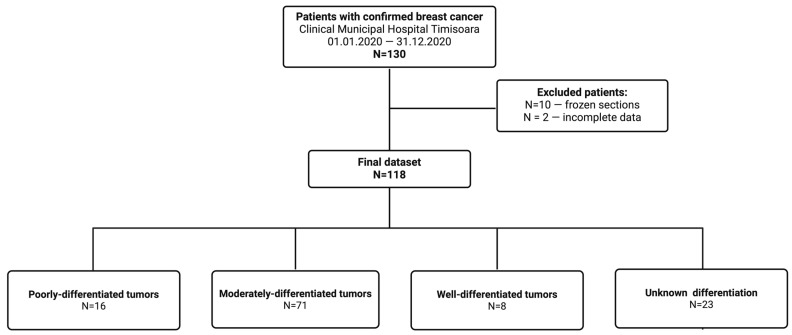
Study flowchart. Created in BioRender. Cindrea, A. (2026) https://BioRender.com/hlh6d1d.

The case selection method, as well as the exclusion criteria, are shown in Figure 1.

### 2.4. Laboratory Technique

All the small biopsies and pathological resection specimens were sent to our department on the same day as the intervention. They were received in formalin; the resection specimens were sectioned into 0.5–1 cm thick slices for better fixation and left for a maximum of 48 h in formalin. After gross description and sampling, the tissue sections were embedded in paraffin, and four micrometer-thick sections were cut using a semi-automated *Leica RM2235* rotary microtome (Leica Biosystems, Nuss Loch, Germany) and displayed on *Super Frost™* microscope slides (St. Louis, MO, USA). For morphological diagnosis, the hematoxylin–eosin technique was primarily used.

The microscopical aspects were recorded for each tumor as follows: architectural pattern of growth, cell type, rate of mitotic figures, necrosis, inflammatory response, calcifications, in situ component, angiolymphatic invasion, and perineural invasion.

For resection specimens, margin status was additionally assessed, and in axillary dissection specimens, each lymph node was examined separately.

Immunohistochemical staining for ER, PR, HER2, and Ki-67 was performed on the Leica Bond-Max, Leica Biosystems, Newcastle, UK automated platform using the standardized laboratory protocols recommended by the manufacturer in use during the study period. All the antibodies and reagents for immunohistochemical staining were purchased from Leica Biosystems, Newcastle, UK. ER and PR positivity were defined as staining in ≥1% of tumor cell nuclei. HER2 was interpreted using the conventional 0/1+/2+/3+ scoring system, with 0 and 1+ considered negative, 3+ considered positive, and 2+ considered equivocal. Ki-67 was assessed as the percentage of positively stained invasive tumor cell nuclei.

### 2.5. Statistical Workflow

Data analysis was performed in R version 4.5.1, R Foundation for Statistical Computing, Vienna, Austria, using the following packages: *dplyr*, *effectsize*, *forcats*, *ggplot2*, *ggsurvfit*, *lubridate*, *patchwork*, *purrr*, *rstatix*, *readr*, *stringr*, *survival*, and *tidyr*. Plots were generated using ggplot-based functions, and multi-panel figures were assembled when needed.

The primary outcome of the study was overall survival (OS), defined as the time from diagnosis to death from any cause.

Descriptive statistics were calculated for all variables. Continuous variables are presented as median (interquartile range, IQR), while categorical variables are reported as counts and percentages. Overall survival was analysed using Kaplan–Meier methods, with time calculated from the diagnosis date to death from any cause; patients without a recorded death were censored at the study cutoff. Because diagnosis timing was available at the month level, index dates were assigned using the recorded diagnosis month (day imputed as the first of the month), and survival time was expressed in months. Survival curves were compared between groups using the log-rank test. To assess differences in survival while accounting for age, Cox proportional hazards models were fitted and reported as hazard ratios (HRs) with 95% confidence intervals (CIs). To improve model stability, treatment was collapsed into no systemic treatment versus any treatment, and histologic grade was collapsed into grade I–II versus grade III. The proportional hazards assumption was assessed using Schoenfeld residuals. Due to the limited number of events, the maximum number of covariables used in the models did not exceed 5.

*p* values below 0.05 were considered statistically significant, and all tests were two-tailed.

### 2.6. Ethics

The study was conducted in accordance with Romanian legislation and the ethical principles set out in the Declaration of Helsinki. Upon admission, patients routinely sign a general informed consent permitting the use of anonymized medical data for academic purposes. Nevertheless, because of the retrospective nature of the study and the exclusive use of existing clinical and pathological data, the requirement for additional informed consent was waived. Histological processing was performed in accordance with the recommendations of the Romanian Ministry of Health and applicable international protocols. Ethical approval for the study was granted by the Ethics Committee of Timișoara Municipal Emergency Clinical Hospital (Institutional Review Board Statement No. E-4249/15 October 2025).

## 3. Results

### 3.1. Descriptive Statistics

The median age at diagnosis for the patients was 62 years, as shown in Table 1. There were 2 women under 35 years, 17 in the 35–50 years old group, 73 in the 51–70 years old group, and 26 over seventy years old. For cases where the information was available, tumor location, types of therapy, surgical interventions, histopathological types of tumors, in situ components (ductal carcinoma in situ-DCIS and lobular carcinoma in situ-LCIS), and molecular subtypes are presented, reported as proportions of the total cohort.

Among all patients, 49 were diagnosed by core needle biopsy (CNB); of these, 24 subsequently underwent mastectomy and one underwent sectorectomy in our institution, with or without neoadjuvant therapy. Five patients were diagnosed by incisional biopsy, followed by mastectomy in two cases, while seven were diagnosed by excisional biopsy/lumpectomy, followed by mastectomy in four cases. Overall, 18 patients underwent sectorectomy, including three who later required mastectomy, 40 underwent mastectomy, and one underwent axillary dissection.

From the 118 cases, 12 presented no clinical data on the location, 30 were described only as right breast tumors, 25 only as left breast tumors, 27 as upper exterior quadrant of which 12 right and 15 left, 11 as central of which 6 right and 5 left, 6 as lower interior quadrant of which 1 right, 5 left and 5 as lower exterior quadrant of which 1 right and 4 left.

In total, 70 mastectomies and 19 sectorectomies were performed, including 3 completion mastectomies after initial sectorectomy. Most procedures were carried out after neoadjuvant therapy, at an interval of 5–12 months from diagnosis, with a median time to surgery of 8.33 months.

Of the patients who underwent surgical treatment, 53 received neoadjuvant therapy. Specifically, 46 received neoadjuvant chemotherapy, with or without HER2-targeted therapy; 3 received neoadjuvant endocrine therapy; 1 received combined chemotherapy and endocrine therapy; 2 received combined chemotherapy and immunotherapy; and 1 received a multimodal regimen consisting of chemotherapy, endocrine therapy, and radiotherapy.

**Figure 2:**
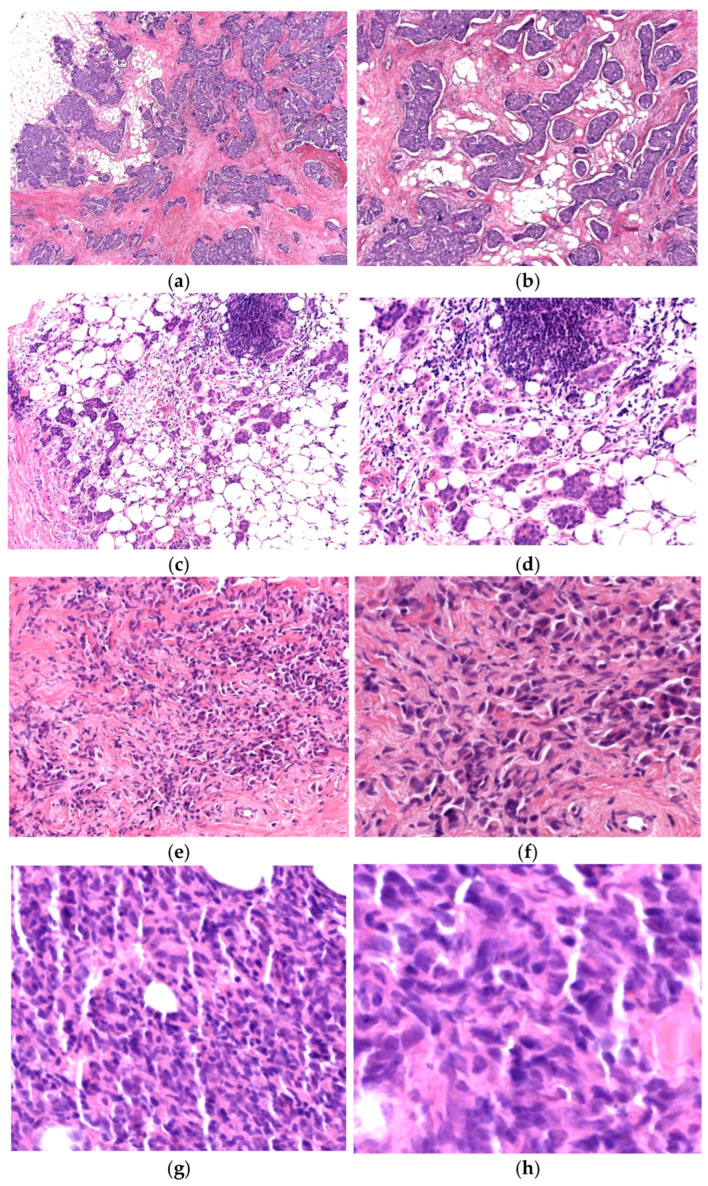
Invasive breast carcinoma NST at 4× (**a**) and 10× (**b**) magnification. Invasive breast carcinoma, moderately differentiated at 10× (**c**) and 20× (**d**) magnification. Invasive lobular carcinoma at 20× (**e**) and 40× (**f**) magnification and non-Hodgkin lymphoma at 40× magnification (**g**) and 60× magnification (**h**).

Almost all the tumors (117) were carcinomas, and one was a non-Hodgkin lymphoma. Most of the carcinomas were invasive NST type, followed by lobular and special types, as depicted in Figure 2.

Scoring of the tumors was made using WHO (World Health Organisation) criteria for breast tumors, assessing glandular formation, nuclear pleomorphism, and mitotic activity, each of them being given a score from 1 to 3. The three values are added together to produce a score of 3–9, to which grades are assigned as follows: 3–5 points = grade 1, well differentiated; 6–7 points = grade 2, moderately differentiated; and 8–9 points = grade 3, poorly differentiated.

**Figure 3:**
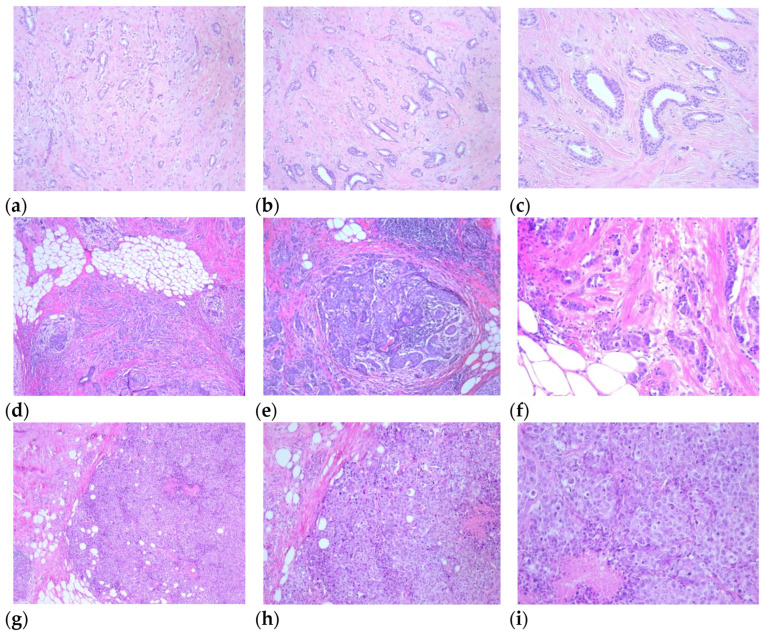
Well-differentiated breast carcinoma at 4× (**a**), 10× (**b**), and 20× (**c**) magnification. Moderately differentiated breast carcinoma at 4× (**d**), 10× (**e**), and 20× (**f**) magnification. Poorly differentiated breast carcinoma at 4× (**g**), 10× (**h**), and 20× (**i**) magnification.

Based on the Nottingham score, of the carcinomas, 95 were graded as follows: 8 tumors were well differentiated, 71 moderately differentiated, and 16 poorly differentiated, as presented in Figure 3.

The molecular subtypes of breast cancer are of utmost importance in the treatment of the disease. Breast tumors are grouped into four basic subgroups according to these markers, luminal A (tumors with either ER or PR positivity, and HER2 negativity), luminal B (tumors with either ER or PR positivity, and HER2 positivity), HER2 positive (tumors with ER and PR negativity, and HER2 positivity), triple negative (tumors with ER, PR, HER2 negativity).

**Figure 4:**
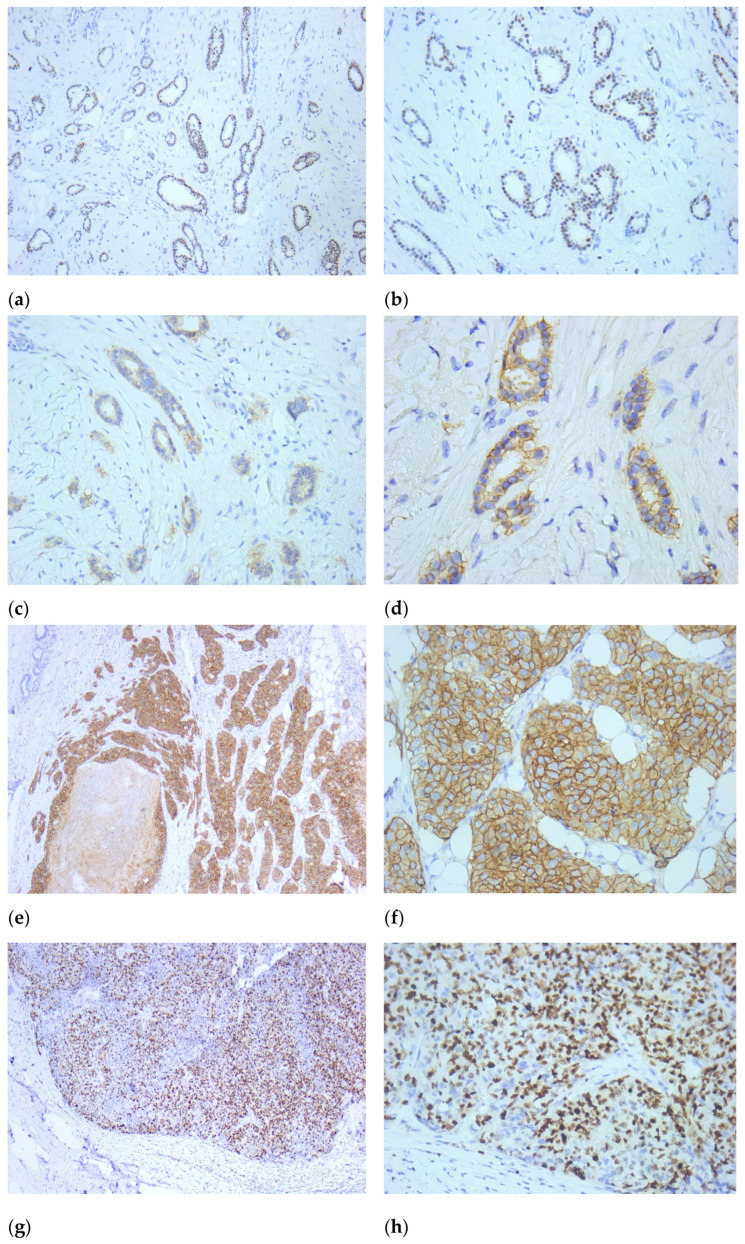
Estrogen receptor staining in a well-differentiated breast carcinoma (4×, (**a**); 10×, (**b**)). Equivocal (2+) HER2 in the same tumor (10×, (**c**); 20×, (**d**)). Positive (3+) HER2 in a moderately differentiated breast carcinoma (10×, (**e**); 20×, (**f**)). Ki-67 in a poorly differentiated breast carcinoma (10×, (**g**); 20×, (**h**)).

Seventy-four carcinomas had immunohistochemistry performed in our laboratory for estrogen receptors, progesterone receptors, HER2 protein, and Ki67, with unknown molecular subtype for the rest. The results were the following: 46 were of luminal B type, 24 of luminal A type, 4 HER2 positive, and one triple negative. Of the 8 well-differentiated tumors, 6 were further tested in our department, with a luminal A profile. Of the 71 moderately differentiated tumors, 59 were further tested in our department, 19 were luminal A, 36 luminal B, and 4 HER2 positive. Of the 16 poorly differentiated tumors, 10 were further tested in our department, with 9 luminal B and one luminal A profile, as presented in Figure 4.

The complete pathological stages correlated to the neoadjuvant therapy are presented in Table 2.

Of the total, 53 patients underwent neoadjuvant therapy, 10 with sectorial mastectomies and the rest with total mastectomies. 17 of the patients with complete pathological response, 7 with stage IA, 7 with stage IIA, 9 with stage IIB, 8 with stage IIIA, 2 with stage IIIB, 3 with stage IIIC. 33 of the patients had no NAT, as presented in Table 2, with age correlations.

Thirty-three of the patients had no NAT, 9 of them with sectorial mastectomies and the rest with total mastectomies, 3 of the patients with complete pathological response, 6 with stage IA, 9 with stage IIA, 8 with stage IIB, 5 with stage IIIA, one with stage IIIB, and one with stage IIIC.

Over a period of five years, 31 of the patients died of the disease (26.27%), 30 with breast carcinomas and one with non-Hodgkin lymphoma, 18 of them in less than a year from the diagnosis, 12 over 70 years of age, 17 between 51 and 70 years of age, and 2 less than or equal to 50 years of age. 28 of the cases had no surgical procedures registered in our database. As for the rest, 9 underwent chemotherapy, 9 had nodal metastases (two pT1, five pT2, one pT3, one pT4), 4 were pT4, one pT0, three pT1, five pT2, three pT3. Twenty-three tumors were moderately differentiated, 5 were poorly differentiated, and 3 were well differentiated, with unknown differentiation for the rest.

Most of the patients died in the first year after the diagnosis. Almost a third of the patients underwent neoadjuvant therapy, and 11% had surgical interventions. 6 patients had stage III tumors (4 of them treated with neoadjuvant chemotherapy), 5 had stage II tumors (2 of them treated with neoadjuvant chemotherapy), one stage I tumor, and one stage 0. Two women died of heart disease, one woman had double neoplasia, and the other died because of tumor progression with metastases.

In the first year of follow-up, 18 patients died; 17 deaths were attributed to disease progression and one to heart disease. Of these patients, two were aged 46 years, eight were 51–70 years old, and eight were older than 70 years. Only four underwent surgical intervention, including three after neoadjuvant therapy (two with stage III disease and one with stage II disease) and one with stage IIIC disease. Most tumors in this subgroup were moderately differentiated carcinomas of the luminal B subtype.

### 3.2. Survival Analysis

**Figure 5:**
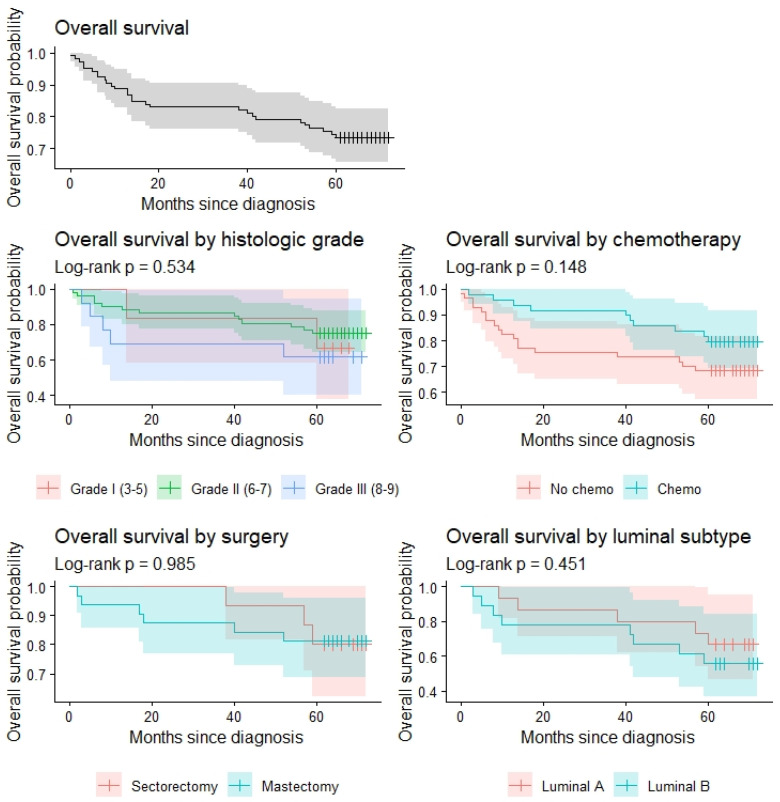
Kaplan-Meier survival curves.

The overall survival curve for the entire cohort and survival curves relative to the intervention, neoadjuvant therapy, luminal type, and histologic grading of the tumors are depicted in Figure 5. Survival distributions were compared using the log-rank test. Table 3 presents the survival rates for each year that has passed, with their corresponding 95% CI.

**Figure 6:**
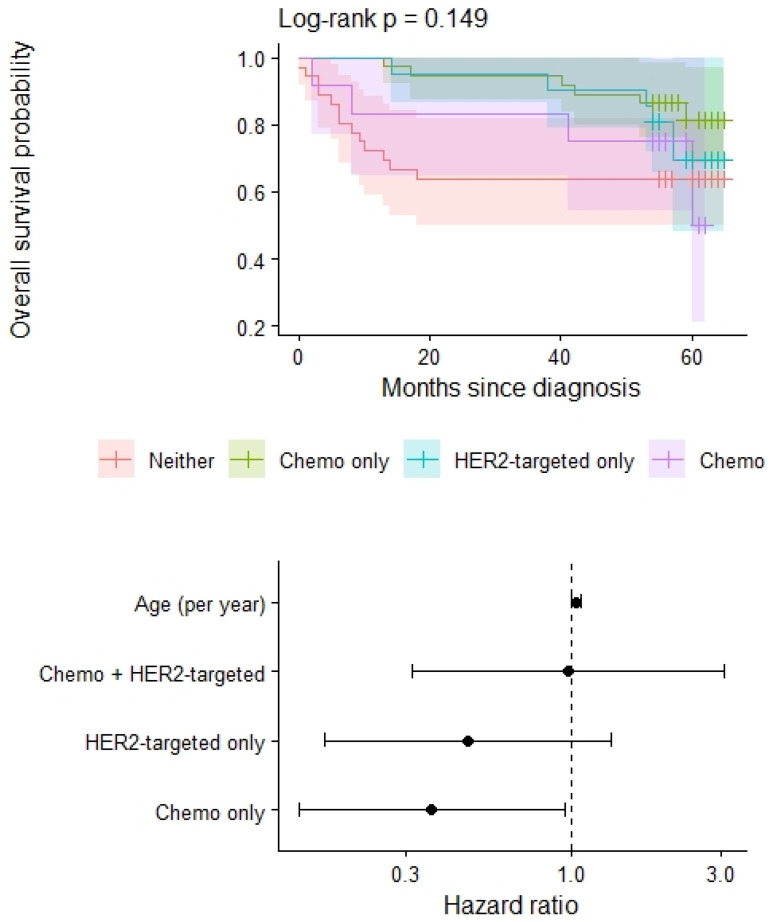
Kaplan-Meier survival curves and forest plot for the Cox proportional hazards model. In the forest plot, hazard ratios are shown as point estimates (dots), while the associated 95% confidence intervals are represented by horizontal line segments.

In an age-adjusted Cox proportional hazards model (Figure 6) including treatment grouping (neither therapy as the reference), older age was associated with a higher risk of death (HRs 1.04 per year; 95% CI 1.00–1.08; *p* = 0.038). Compared with patients receiving neither neoadjuvant chemotherapy nor HER2-targeted therapy, those treated with neoadjuvant chemotherapy alone had a significantly lower hazard of death (HRs 0.36; 95% CI 0.14–0.96; *p* = 0.041). The hazard of death was also lower among patients receiving HER2-targeted therapy alone, although this did not reach statistical significance (HRs 0.47; 95% CI 0.17–1.34; *p* = 0.159). No difference in mortality risk was observed for patients receiving combined neoadjuvant chemotherapy and HER2-targeted therapy relative to the reference group (HRs 0.98; 95% CI 0.32–3.07; *p* = 0.979). Overall, the model demonstrated modest discriminative performance (concordance 0.68), and the global test for model fit suggested borderline evidence of an association between treatment group and survival (likelihood ratio test *p* = 0.04). About 32% of all the women in the group treated with neoadjuvant therapy have reached complete pathological response. Out of those, 15 were treated with mastectomies and 2 with sectorectomies, with most of them (98%) still alive at 5-year follow-up.

In the multivariable Cox proportional hazards model including age, treatment exposure, histologic grade, molecular subtype, and surgical treatment status, grade III tumors were independently associated with worse overall survival compared with grade I–II tumors (HR 9.21, 95% CI 1.19–71.05, *p* = 0.033). Age, treatment exposure, subtype, and surgery were not significantly associated with overall survival in the adjusted model. The proportional hazards assumption was not violated (global Schoenfeld test *p* = 0.56). Due to the relatively small number of events, the multivariable model should be interpreted with caution and results considered exploratory.

## 4. Discussion

Breast cancer is a worldwide health issue encompassing a spectrum of molecular subtypes and clinical presentations, each with distinct prognostic implications and treatment responses [23]. The incidence of the disease is rising in younger women [24], but the most affected are still women over 50 years old [25], with few cases under 35 years of age. The age distribution in our study is also consistent with this description, 83.8% of the patients being postmenopausal (61.8% in the 51–70 age group) and only 16.1% under 50 years old. Breast tumors arise from an association of genetic and non-genetic factors [26], widely studied and with particular features in premenopausal versus postmenopausal women [27].

In the current study, age at diagnosis was one of the variables most strongly associated with overall survival, with younger women having a more favorable prognosis. This relationship was also influenced by other tumor-related characteristics, particularly the distinct biological features of the tumors [6].

The majority of tumors were located in the upper quadrant, especially the right upper quadrant, in accordance with the published data and with no statistically relevant correlation with overall survival. Though primary tumor location has been studied as a potential prognostic factor, its relevance still remains to be determined [28].

The most commonly used type of diagnostic intervention for our patients was core needle biopsy, the gold standard for diagnosing breast lesions [29]. Though discouraged [30], frozen section is still a diagnostic procedure in less than 6% of the cases in our institution. Core needle biopsy is efficient for both breast tumors and axillary lymph node evaluation [31].

An additional important procedure in breast cancer staging is SLNB, indicated in specific cases, which can avoid axillary lymph node dissection [32]. The most frequent complications are represented by lymphedema, paresthesia, range-of-motion restriction, and pain in the arm ipsilateral to the lymph node dissection [33]. In our current practice, this type of intervention is exceptional, absent in all the presented cases.

The surgical therapeutic intervention of choice was mastectomy with axillary dissection, irrespective of the stage of the tumor and age of the patient, representing more than 80% of interventions for diagnosed and/or managed cases in our study. The majority of women with complete pathological response (88%) had mastectomies, and the rest had sectorectomies. All cases were accompanied by axillary dissection. Botezatu et al. [34], in a recent study conducted in Bucharest, Romania, concluded that the standard surgical procedure for breast cancer is modified radical mastectomy. Even though breast-conserving surgery (BCS) is the preferred treatment for early unifocal breast cancer [35,36], it is relatively rarely performed in our institution. Although breast conserving therapy is becoming more and more popular, mastectomy is preferred, especially when it comes to locally advanced disease, inflammatory carcinoma, or multicentric disease [37].

BCS with adjuvant radiotherapy is considered the gold standard approach for patients with early-stage breast cancer [38]. The efficacy and long-term advantages of conservative surgery are defined by overall and disease-free survival rates equivalent to those of mastectomy [39]. When it comes to differentiating between molecular subtypes, data from the literature are partially contradictory, indicating that there is a significantly better prognosis in patients with luminal and triple-negative subtypes, with no significant better prognosis in HER2-positive cases [40], while other studies suggest that it improved overall survival, particularly in HER2-enriched and triple-negative subtypes [41]. The literature remains inconsistent, with some studies suggesting that breast-conserving surgery may be more effective than mastectomy in younger patients, while others report a higher rate of local recurrence in this group [42,43].

More than half of the patients with mastectomies and sectorectomies (61.6%) were treated with neoadjuvant therapy, proven as efficient as adjuvant therapy with superior advantages in inoperable, as well as operable disease [44]. One in three patients treated with NAT achieved a pathological complete response, a proportion higher than that reported in other studies, like Haque et al. [45] or Houssami et al. [46], where one in five patients presented no residual tumor. This difference is clinically relevant and may reflect the influence of multiple variables, among which tumor bed assessment is particularly important from a pathological perspective and deserves further study. Most of the patients with pCR, irrespective of age, were still alive at 5 years of follow-up. Some studies showed that pCR predicts long-term survival even in cases of high-risk breast cancer [47].

Though therapy response is dependent on the treatment protocols, studies show that most regimens have very similar efficacy profiles as first- and subsequent-line therapies [48]. Complete pathological response can be related to multiple variables, and the results of our study are comparable to those of pCR for BRCA-mutated cases [49]. There are studies describing favorable pCR in locally advanced disease irrespective of tumor size and type of chemoprotocol [49] and in younger women with breast cancer [50], although younger patients and those with stage IIIB and IIIC are more likely to develop distant metastasis [51].

Our study aligns with other findings suggesting that patients who achieve a pCR after NAC may have better overall survival compared with matched patients having only a partial pathological response [52].

Meta-analysis combining data for complete pathological response rates and survival outcomes, divided into disease-free survival, event-free survival, relapse-free survival, and overall survival [53], is needed to evaluate the differentiated effects of pCR.

Extensive literature supports the finding that pCR is linked to a better prognosis, indicating that it is an independent prognostic factor regardless of staging or HR expression [54]. 

The correlation with molecular subtypes was also extensively studied. Practically, HER2-positive cancers are the ones that most frequently reach a complete pathological response among TNBC [17,55].

The percentage of patients undergoing neoadjuvant chemotherapy in our study decreased as age increased, possibly linked to the impact of associated comorbidities on prognosis, response to therapy, adverse events, and overall survival. Some studies have concluded that carefully selected patients aged ≥70 years can safely receive NAC with substantial clinical benefit, their clinical outcomes being similar compared to younger women, suggesting that shorter regimens may be appropriate [56].

The correlation between pCR and survival outcomes varies among different breast cancer molecular subtypes. In TNBC, achieving pCR is strongly associated with a significant reduction in recurrence risk and improved OS, while in hormone receptor-positive/HER2-negative breast cancer, the relationship between pCR and survival is more complex, as endocrine responsiveness plays a crucial role in long-term prognosis, often independent of the pCR status [57].

There are multiple types of neoadjuvant therapies and combinations. The most frequently recommended ones are chemotherapy, endocrine therapy, and HER2-targeted therapy [58]. The treatment schemes are thoroughly defined in clinical practice guidelines, depending on tumor histology, grade, stage, and estrogen, progesterone, and human epidermal growth factor receptor 2 (HER2) expression [59]. Wang et al.l show that postmenopausal HR-positive breast cancer patients after NAT may have better tumor response than those after NET (neoadjuvant endocrine therapy) [60]. In our study, of the 53 patients treated prior to the intervention, 43 (86.8%) received NAT (+/− HER2 targeted therapy), 3 (5.6%) received NET, one received combined neoadjuvant chemo- and hormonal therapy, two received combined chemo- and immunotherapy, and one received combined chemo-, hormonal, and radiotherapy.

As is the case with our study, invasive breast carcinoma is by far the most common type of breast cancer, followed by lobular carcinoma [61]. Each case is further tested for a standard panel of four markers, for therapeutic purposes. In our institution, 74 cases were tested for estrogen and progesterone receptors, HER2, and Ki67 index using immunohistochemistry. Only a limited number of the other cases had information about the molecular profile. Hormone-dependent invasive carcinoma of no special type represented most of the tumors, with luminal B as the most common molecular subtype, for well- and poorly differentiated tumors.

Pathological staging of the tumors was done according to the AJCC/TNM criteria [62], taking into consideration tumor size and invasion, lymph node metastasis, and distant metastasis. The distribution of the patients in the group with NAT is the following: of the 53 patients, 32% were stage 0, 13.2% stage IA, 13.2% stage IIA, 16.9% stage IIB, 15% stage IIIA, 3.7% stage IIIB, 5.6% stage IIIC. Of the 33 patients without neoadjuvant therapy, 3% were stage 0 (DCIS), 18.1% stage IA, 27.2% stage IIA, 24.2% stage IIB, 15.1% stage IIIA, 3% stage IIIB, and 3% stage IIIC. 32% of the participants had a complete pathological response, with a higher fraction in premenopausal women.

Over a period of 5 years, one in three women died of the disease, associated complications, or secondary malignancies, with most of the patients (58%) dying in the first year after the diagnosis. In the first year, 44.4% of the deaths occurred in women over 70 years old. More than half of the patients did not undergo any further interventions in our institution, which can suggest that other factors, like advanced inoperable disease or patient noncompliance, contributed to an early death.

Mortality rate of breast cancer in Romania has an increasing trend and is higher compared to other EU countries [63]. The causes of mortality vary from associated comorbidities or tumor progression to multiple neoplasms, the most common being genitalia, lung, or colorectal cancer [64]. Some authors conclude that the death rate, especially for older women with breast cancer, is even higher due to associated comorbidities [65].

The analysis of demographic, socio-economic, lifestyle, and clinical predictors, combined with an evaluation of regional disparities [66], could explain the causes for the higher mortality rate in our country. Frequent late-stage diagnoses [67] might be due to the absence of a functional national screening program [68], restricted access to smaller organized mammography screenings [69], and poor education that makes self-examination difficult [70]. Pilot screening programs took place, with less than 1% of the women between 50 and 74 years participating [71], and an ongoing national screening program is in progress. The absence of a National Cancer Registry limits the monitoring of cancer incidence, the evaluation, and control of the disease [72]. Although due to poor spatial accessibility to health-care services, rural residents are less likely to receive early and appropriate diagnoses and effective treatments [73], some studies describe even higher mortality rates in women in urban areas, this being likely to indicate other related social and structural factors that still need investigation [74].

Cancer management should be a multidisciplinary approach to minimizing recurrence and reducing treatment-associated morbidity [75]. Socioeconomic factors should be addressed and improved, given their important relationship with the prevalence of advanced disease [76]. New therapeutic strategies and participating in national screening programs help reduce breast cancer-related mortality [77].

Several limitations of this study must be acknowledged. The retrospective design and single-center setting limit external validity. Missing immunohistochemical data may have affected molecular classification and weakened subtype-based analyses. Furthermore, the dataset lacked detailed information on neoadjuvant regimens, including drug composition, number of administered cycles, and the use of HER2-targeted treatment, because these data were inconsistently available in the electronic medical records. Key clinical confounders, including comorbidities and performance status, were not systematically available. An important source of selection bias is that 28 of the 31 patients who died had no registered surgical intervention in our institution; this likely reflects a subgroup with more advanced disease, non-compliance with treatment, or referral-related loss from institutional records, and may have disproportionately influenced survival outcomes. In addition, potentially important unmeasured confounders, including socioeconomic barriers to access, delays in presentation, and incomplete staging information in non-operated patients, may have influenced both treatment pathways and overall survival, thereby limiting causal interpretation of the observed associations.

Despite these limitations, this study has important strengths. Firstly, it reflects real-world practice in a Romanian tertiary center, includes a five-year follow-up period, and incorporates mortality record verification, thereby providing relevant longitudinal survival data in a setting where institutional outcome reports remain scarce. In addition, the integration of pathological characteristics, treatment data, response to neoadjuvant therapy, and survival analysis offers a clinically meaningful picture of local breast cancer management.

Future studies should build on these findings through multicenter, prospectively collected cohorts with standardized recording of patients’ data. Such work would improve external validity and support more accurate prognostic modeling in the Romanian population.

## 5. Conclusions

In conclusion, this study identified several clinicopathological and treatment-related factors associated with overall survival in our cohort. Beyond the institutional findings, the results may also reflect broader challenges still affecting breast cancer care in Romania, including late-stage presentation and barriers to completion of multidisciplinary treatment. Addressing these gaps through earlier diagnosis, better continuity of care, and improved access to guideline-concordant therapy may help reduce survival disparities at the national level.

## Figures and Tables

**Table 1:** Descriptive statistics of the patient cohort.

Variable	All Patients (N = 118)	Variable	All Patients (N = 118)
Age (years) ^a^	62 (53–70)	Nottingham Grade I (3–5 points)	6 (7.3%)
Left-sided tumor	54 (48.2%)	Nottingham Grade II (6–7 points)	61 (74.4%)
Exulcerated lesion	4 (3.4%)	Nottingham Grade III (8–9 points)	15 (18.3%)
CQ	13 (23.6%)	DCIS	3 (2.9%)
LOQ	3 (5.5%)	In_situ	1 (1%)
LIQ	4 (7.3%)	Lobular	7 (6.8%)
UOQ	28 (50.9%)	Metastasis	2 (1.7%)
Biopsy	61 (51.6%)	Mucinous	2 (1.9%)
Excision	3 (2.7%)	NST	80 (77.7%)
Mastectomy	33 (29.5%)	Papillary	3 (2.9%)
Sectorectomy	15 (13.4%)	Other	4 (3.9%)
Chemotherapy	49 (41.5%)	Described in-situ component	23 (19.5%)
Hormone-therapy	5 (4.2%)	Luminal A-like	15 (12.7%)
HER-2	14 (11.86%)	Luminal B-like	45 (38.1%)

Variables are presented as absolute value (percentage out of the total number of entries); ^a^—median (inter quartile range). Abbreviations: CQ, central quadrant; LOQ, lower outer quadrant; LIQ, Lower inner quadrant; UOQ, upper outer quadrant; DCIS, in situ ductal carcinoma; NST, no special type.

**Table 2:** Distribution of neoadjuvant therapy and stage by age group.

Variable	All Patients (*n* = 118)	<35 Years (*n* = 2)	35–50 Years (*n* = 17)	51–70 Years (*n* = 73)	>70 Years (*n* = 26)
Age median (IQR)	62 (53–70)	33 (32.5–33.5)	46 (43.8–48)	61.5 (56.8–64.2)	75 (73–80.5)
**NAT**	**53 (44.9%)**	**2 (100.0%)**	**10 (58.8%)**	**34 (46.5%)**	**7 (26.9%)**
Stage 0	17 (32.1%)	1 (50.0%)	5 (50.0%)	9 (26.5%)	2 (28.6%)
Stage IA	7 (13.2%)	1 (50.0%)	3 (30.0%)	1 (2.9%)	2 (28.6%)
Stage IB	-	-	-	-	-
Stage IIA	7 (13.2%)	-	1 (10%)	6 (17.6%)	-
Stage IIB	9 (17%)	-	-	9 (26.5%)	-
Stage IIIA	8 (15.1%)	-	1 (10%)	5 (14.7%)	2 (28.6%)
Stage IIIB	2 (3.8%)	-	-	2 (5.9%)	-
Stage IIIC	3 (5.7%)	-	-	2 (5.9%)	1 (14.3%)
**No NAT**	**65 (55.1%)**	**-**	**7 (41.2%)**	**39 (53.4%)**	**19 (73.1%)**
Stage 0	3 (4.6%)	-	1 (14.3%)	-	2 (33.3%)
Stage IA	18 (27.7%)	-	4 (57.1%)	10 (25.6%)	4 (27.3%)
Stage IB	-	-	-	-	-
Stage IIA	11 (16.9%)	-	2 (28.6%)	9 (23.1%)	-
Stage IIB	16 (24.6%)	-	-	7 (17.9%)	9 (45.5%)
Stage IIIA	11 (16.9%)	-	-	9 (23.1%)	2 (13.6%)
Stage IIIB	1 (1.5%)	-	-	1 (2.6%)	-
Stage IIIC	5 (7.7%)	-	-	3 (7.7%)	2 (13.6%)

Data are presented as *n* (%) unless otherwise indicated. Percentages in the NAT and No NAT rows are column percentages. Percentages in stage rows are calculated within the corresponding NAT stratum and age group. Abbreviations: IQR—interquartile range; NAT—neoadjuvant chemotherapy.

**Table 3:** Survival rates for the entire population.

Years	Months	OS	95% CI
1	12	88.7%	82.8–94.9%
2	24	83%	76.1–90.5%
3	36	83%	76.2–90.5%
4	48	79%	71.9–87.4%
5	60	72.8%	64.2–82.5%

Abbreviations: OS—overall survival; 95% CI—95% confidence interval.

## Data Availability

The data that support the funding for this study are available from the corresponding author upon reasonable request.

## Abbreviations

ALD	axillary lymph node dissection
AJCC	American Joint Cancer Committee
BCS	breast conserving surgery
BRCA	Breast Cancer gene
CAP	College of American Pathologists
CIs	confidence intervals
CNB	core needle biopsy
CQ	central quadrant
DCIS	ductal carcinoma in situ
ER	estrogen receptor
EU	European Union
HR	hormone receptors
HRs	hazard ratios
HER2	human epidermal growth factor receptor
IQR	interquartile range
LCIS	lobular carcinoma in situ
LIQ	lower inner quadrant
LOQ	lower outer quadrant
NAT	neoadjuvant therapy
NCCN	National Comprehensive Cancer Network
NET	neoadjuvant endocrine therapy
NST	no special type
OS	overall survival
pCR	pathological complete response
PR	progesterone receptor
RCB	Residual Cancer Burden
SLND	sentinel lymph node dissection
TNBC	triple negtive breast cancer
TNM	tumor-node-metastasis
UOQ	upper outer quadrant
WHO	World health Organisation
